# Innocent lives lost and saved: the importance of blood transfusion for children in sub-Saharan Africa

**DOI:** 10.1186/s12916-014-0248-5

**Published:** 2015-02-02

**Authors:** Walter H Dzik

**Affiliations:** Blood Transfusion Service, Massachusetts General Hospital, Boston, MA 02114 USA

**Keywords:** Transfusion, sub-Saharan Africa, Severe anemia

## Abstract

Severe anemia in children is a leading indication for blood transfusion worldwide. Severe anemia, defined by the World Health Organization as a hemoglobin level <5 g/dL, is particularly common throughout sub-Saharan Africa. Analysis of data from the Fluid Expansion as Supportive Therapy (FEAST) trial offers new insights into the importance of blood transfusion for children with severe anemia. The principal findings of this analysis include the observations that life-threatening anemia in children is a frequent presenting condition in East Africa; that delays in transfusion therapy are lethal; and that inadequate transfusion is probably more common than currently recognized. The findings of this new study highlight the need for changes in blood inventory management in sub-Saharan hospitals and the need for more research on transfusion therapy for children in peril.

Please see related article: http://dx.doi.org/10.1186/s12916-014-0246-7

## Background

In sub-Saharan Africa (SSA), the death of a child is an everyday tragedy. The sorrow is compounded by the fact that most children die not as a result of complex congenital abnormalities, from malignancy, or from chronic incurable diseases, but rather from completely treatable and reversible conditions such as severe malarial anemia. Simply stated, blood transfusion therapy for children in SSA is a matter of life and death.

In recent years, national blood transfusion programs in SSA have made steady and significant progress [1]. Supported by government financing and through external aid from programs such as the President’s Emergency Plan for AIDS Relief (PEPFAR), national initiatives for volunteer donor blood collection, viral testing, and distribution to hospitals continue to advance. Nevertheless, even in well-run programs, supply falls short of demand, and blood donations per 1,000 citizens in low-income countries are far below that seen in wealthy nations [2]. The pattern of blood usage is also changing in SSA with growing demands for blood products to support cancer care and to treat the burden of trauma brought on by increasing road traffic accidents, burns from open fires, and human violence [3]. Nevertheless, children with severe anemia remain one of the most important categories for blood transfusion in SSA, and high-quality information focused on pediatric blood transfusion is both useful and welcome.

In a research article published in *BMC Medicine,* Sarah Kiguli and colleagues present an important and data-rich report from East Africa that details the use and the outcomes of blood transfusion in children [4]. The report analyzes data from the Fluid Expansion as Supportive Therapy (FEAST) trial, which is a previously published randomized controlled trial of volume loading in children who presented with severe febrile illness to six hospitals in three East African nations during the period 2009 to 2011 [5]. Enrollment required that children exhibit respiratory distress or impaired consciousness plus signs of impaired tissue perfusion. As a result, the FEAST trial focused on very sick children presenting for emergency care, and this is reflected by the fact that, overall, one in ten children enrolled in the study died within the first 48 hours.

## What are the main findings of this new report?

While there are many lessons in the data, readers will want to take away three major findings.

### Life-threatening anemia in sick children is extremely common

Hemoglobin testing was done on presentation to hospital for over 3,000 children who met enrollment criteria for the FEAST trial. Among these, a staggering 33% were severely anemic, defined as having a hemoglobin level <5 g/dL. In the majority of cases the anemia was a consequence of malaria and thus completely correctable with appropriate therapy. Indeed, severe anemia is the most common of three major sub-syndromes of acute *P. falciparum* malaria and when accompanied by lactic acidosis is highly associated with a fatal outcome [6]. The robust data set reported by Kiguli *et al.* provides the best measure to date of the magnitude of life-threatening anemia in children presenting to hospitals in East Africa.

### Any delay in blood transfusion is lethal

The single most striking finding of Kiguli *et al.* is the absolute urgency of severe anemia in children. Consider the following data taken from this report: Among children with only moderate anemia (hemoglobin 5 to 7 g/dL), those transfused within 8 hours of arrival had a 5% mortality which was not different from the 4% mortality of those transfused after 8 hours. However, among children presenting with severe anemia (hemoglobin <5 g/dL), the mortality among those transfused within 8 hours of arrival was also 4%, but the mortality among those for whom transfusion was delayed by more than 8 hours was 52%. Indeed, 90% of these deaths occurred within just 2.5 hours of arrival, and 100% of the deaths were within the first five hours.

### Inadequate transfusion (under-treatment) is probably common

Given the limited blood resources throughout SSA, current guidelines focus on transfusion support for the most severely anemic individuals and recommend 10 mL/kg of packed red blood cells (or 20 mL/kg of whole blood). While these guidelines conserve use of a precious resource, they also likely leave a substantial number of children in a condition of relative under-treatment. In the report of Kiguli *et al.*, nearly one-quarter of the children who received an initial transfusion according to guidelines ultimately received additional transfusions. Not surprisingly, these repeat transfusions were associated with those who presented with the most profound levels of anemia, with extreme tachycardia, or in coma suggesting that the current standard is not a sufficient dose for those at greatest clinical risk.

## Unanswered questions and next steps

The work of Kiguli *et al.* presents disturbing and provocative information on the number of innocent lives lost to anemia in SSA. The findings also point the way towards achieving better outcomes. The data presented likely underestimate the tragedy of insufficient transfusion support, because there are no published accounts of the frequency with which urgent requests for blood go unfilled for lack of supply - especially in more remote regions of SSA. Nevertheless, for children with severe anemia, this study reminds us, with substantial data, that children are adaptive but are not invincible. Given the findings regarding the high prevalence of severe anemia and the lethal consequences resulting from delays in transfusion, we now have the most robust data yet published to argue convincingly for a resetting of goals for blood stocks throughout the malarial regions of SSA. Safe blood should not be a treatment rationed for life-threatening physiology nor procured at the time of need, but rather must be available on site. In fact, a reserve of emergency Group O packed red cells is standard in hospitals of all wealthy nations and needs to become a target goal for SSA.

For the far larger population of children with moderate anemia, the report of Kiguli *et al.* demonstrates the current uncertainties of treatment that translate directly into a wide variation in transfusion practice. What is needed for these children is more clinical research that will identify which children are in need of transfusion, what the proper dose should be, and what the target goal of therapy is - how much transfusion is appropriate. Such research will be of substantial value to the great number of children in SSA with sickle cell disease or nutritional anemia who are likely to be underserved by current transfusion practice.

An available supply of safe blood represents a fundamental healthcare infrastructure. Efforts underway in SSA to organize national volunteer donor programs and to promote professionals in transfusion medicine should be supported [7]. Research designed to document the safety and efficacy of stored blood, such as the Transfusion in Severe Anemia with Lactic Acidosis trial [8], will be of great value towards promoting an adequate blood supply. Perhaps most importantly, all parties - government, funding agencies, national blood transfusion services, clinicians, religious groups, the media, and the general public - need to reject complacency with the status quo. Only then will innocent lives, stricken with a fully treatable condition, continue to be rescued.
